# Glia Imaging Shows Clinical Utility in Differentiating Parkinson's Disease from Multiple System Atrophy

**DOI:** 10.1002/mds.29078

**Published:** 2022-06-06

**Authors:** Juha O. Rinne, Aurelija Jucaite, Zsolt Cselényi, Lars Farde

**Affiliations:** ^1^ Turku PET Centre University of Turku and Turku University Hospital Turku Finland; ^2^ Department of Clinical Neuroscience Centre for Psychiatry Research, Karolinska Institutet Stockholm Sweden; ^3^ PET Science Centre, Personalized Medicine and Biosamples, R&D, AstraZeneca Stockholm Sweden

1

We recently presented a multicenter positron emission tomography (PET) study on the glia biomarker translocator protein (TSPO) in patients with multiple system atrophy (MSA) and Parkinson's disease (PD).^1^ We found a distinct TSPO pattern for MSA with an elevated signal in the striatum (lentiform nucleus in particular) and cerebellar white matter (with 96% sensitivity and 100% specificity against a clinical MSA diagnosis). However, in the imaging data analysis, we observed a single patient with MSA who deviated in glia pattern across both visual and machine‐learning assessment approaches. Following this observation, we now review this case in detail.

The patient is a 50‐year‐old woman (Fig. 1B, patient 9) diagnosed with possible MSA based on clinical symptoms of bradykinesia, stiffness, dysautonomia (segmental left‐sided hyperhidrosis), and collapse attacks (thought to be attributed to orthostatism). At that time, single‐photon emission computed tomography showed reduced dopamine transporter in the right striatum, especially in the posterior putamen. Brain magnetic resonance imaging (MRI) did not show any remarkable findings. Levodopa treatment was started, and she has had good and sustained response and the dysautonomic features have diminished. She has had no cerebellar or pyramidal signs. At 8 years after the diagnosis, she was included in a clinical trial as a patient with MSA. A two‐level diagnostic procedure was undertaken: MSA diagnosis was established by an investigator at a specialized movement disorders clinic, and the assurance of diagnostic accuracy was performed by blinded independent expert review.^1^ The patient is a mixed‐affinity binder by *TSPO* genotype. However, at the baseline PET examination, the glia pattern (Fig. 1A) did not resemble that of other patients in the MSA group (Fig. 1C), and she was the only patient identified as misclassified compared with the clinical diagnosis. At follow‐up 11 years after the initial diagnosis, the patient was rediagnosed with PD.

**FIG 1:**
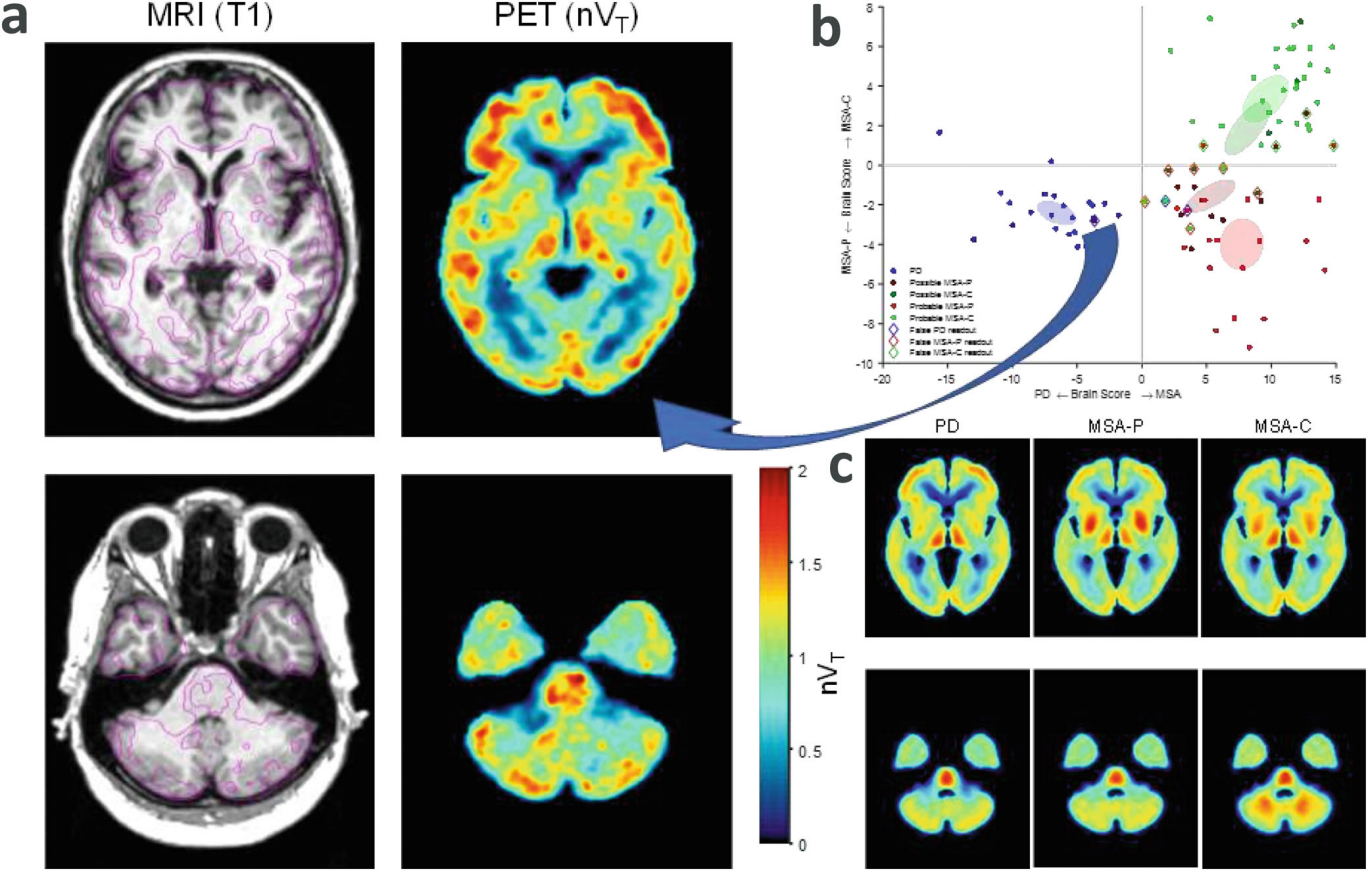
(**A**) Left panel shows horizontal slices of MRI (T1‐weighted) and PET (nV_T_ parametric) images for patient 9 with magenta lines on MRI indicating contours of PET. (**C**) Lower right panel shows median nV_T_ images for patient groups with PD, MSA‐P, and MSA‐C. All images are in MNI template space to facilitate comparison. The location of horizontal slices matches between individual and group images with the upper one cutting through the lentiform nucleus and the lower one through the pons and middle cerebellar peduncle. Normalized cortical binding in patient 9 is similar to PD median levels. Importantly, no MSA‐specific “hot spots,” that is, binding levels reaching or surpassing those in the cortex, in either the lentiform nucleus or in the cerebellar white matter can be seen in patient 9. (**B**) Upper right panel shows the results of diagnostic readout obtained using cross‐validated machine learning. Arched arrow indicates patient 9 in the score scatterplot. For a more detailed description of panels B and C, see Jucaite et al.^1^ MRI, magnetic resonance imaging; MSA‐C, multiple system atrophy–cerebellar; PD, Parkinson's disease; PET, positron emission tomography; nVT, normalized total volume of distribution; MNI, Montreal Neurological Institute; MSA‐P, multiple system atrophy of parkinsonian type.

Based on the clinical course, that is, good levodopa response 15 years after the diagnosis, ability to walk well, diminished dysautonomic features, no pyramidal or cerebellar signs, no antecollis or stridor, and life longevity, this patient with MSA is now confirmed as PD.

## 2 Discussion

This case is an example of the challenge faced by clinicians in the differentiation of early MSA with parkinsonian symptoms from PD. The distinct triad of tremor, bradykinesia, and muscle rigidity describing motor impairment in PD may not always be present. Today, PD is known as a heterogenous disease, presenting in different disease variants. Clinical subtypes can range from a mild subtype, for example, a combination of motor phenotype, response to dopaminergic treatment, and slow progression, to a malignant subtype with broader symptom spectrum, poor response to treatment, and fast progression.^2^ The latter may closely resemble possible MSA. In this case presentation of disease onset with dysautonomia, the symptoms led to a suspicion of MSA.

Clinical diagnostic criteria of MSA have been supported with characteristic signs observed at imaging, for example, patterns of glucose metabolism, changes in dopamine transporter levels, and specific structural changes detected on MRI.^3^ Notably, these imaging modalities have provided evidence on the significant differences between patient groups with PD and MSA. However, they have limitations in the individual case diagnostics, for example, by the need of comparison of individual patient data against group threshold levels ([^18^F]luorodeoxyglucose^4^) or less applicable for the MSA cerebellar subtype (dopamine transporter imaging^5^). Meanwhile, the TSPO pattern in MSA is apparent in each individual case and was observed using different PET systems, with excellent sensitivity and specificity of blinded visual read.^1^ In addition, a differential diagnosis using TSPO imaging is enabled by the absence of an elevated TSPO signal in PD compared with controls, as has been reported repeatedly using different radioligands.^6^, ^7^

The review of clinical diagnostic criteria, disease course, and response to dopaminergic therapy supported by the absence of an MSA‐specific glia pattern supported to change the patient's diagnosis form MSA to PD.

This case illustrates the clinical utility of glia imaging in the differential diagnostics of MSA and PD. If early glia imaging would be considered, diagnosis could be established much earlier. Importantly, the glia imaging could help to make the diagnosis at the individual case level and even by a visual inspection of brain TSPO images.

## 3 Author Roles

(1) Research Project: A. Conception, B. Organization, C. Execution; (2) Statistical Analysis: A. Design, B. Execution, C. Review and Critique; (3) Manuscript: A. Writing of the First Draft, B. Review and Critique.

J.O.R.: 1A, 3A, 3B

A.J.: 1A, 3A, 3B

Z.C.: 1A, 2A, 2B, 3A, 3B

L.F.: 1A, 3A, 3B

## Data Availability

The imaging data that support the case presentation are available from the corresponding author upon reasonable request. Individual clinical data is not shared, as protected by GDPR (EU) 2016/679.
